# Titanate-based high-entropy perovskite oxides relaxor ferroelectrics

**DOI:** 10.1038/s41598-024-55402-0

**Published:** 2024-03-12

**Authors:** Ketkaeo Bunpang, Suparat Singkammo, David P. Cann, Natthaphon Raengthon

**Affiliations:** 1 Department of Materials Science, Faculty of Science, Chulalongkorn University, Bangkok, 10330 Thailand; 2 Center of Excellence in Physics of Energy Materials (CE:PEM), Department of Physics, Faculty of Science, Chulalongkorn University, Bangkok, 10330 Thailand; 3 Synchrotron Light Research Institute (Public Organization), Nakhon Ratchasima, 30000 Thailand; 4 Materials Science, School of Mechanical, Industrial and Manufacturing Engineering, Oregon State University, Corvallis, OR 97331 USA; 5 Center of Excellence on Advanced Materials for Energy Storage, Chulalongkorn University, Bangkok, 10330 Thailand

**Keywords:** Electronic properties and materials, Ferroelectrics and multiferroics

## Abstract

Different combinations of monovalent and trivalent A-cations in high-entropy perovskite oxides (HEPOs) were investigated. The multicomponent (A′_0.2_A″_0.2_Ba_0.2_Sr_0.2_Ca_0.2_)TiO_3_ (A′ = Na^+^, K^+^, A″ = Bi^3+^, La^3+^) perovskite compounds were successfully synthesized by solid-state reaction method persisting average cubic perovskite phase. The trivalent cation exhibited distinct effects on local structure, dielectric properties and relaxor ferroelectric behavior. Highly dense ceramics (> 95%), high dielectric constant (~ 3000), low dielectric loss (~ 0.1), and relaxor ferroelectric characteristics were obtained in the compound containing Bi^3+^. The La^3+^ containing compounds revealed lower dielectric constant, higher dielectric loss and linear dielectric behavior. The effect of monovalent cation on the dielectric properties was minimal. However, it affected relaxor ferroelectric behavior at elevated temperatures and conduction behavior at high temperatures. The (K_0.2_Bi_0.2_Ba_0.2_Sr_0.2_Ca_0.2_)TiO_3_ ceramic maintained the relaxor ferroelectric behavior with low P_REM_ at high temperatures suggesting more stable relaxor ferroelectric characteristics than that of the (Na_0.2_Bi_0.2_Ba_0.2_Sr_0.2_Ca_0.2_)TiO_3_. Moreover, between these two compounds, the homogeneous electrical characteristics could be obtained from the compound consisting of K + and Bi + at A-site. This study suggests that tuning the chemical composition, particularly choosing appropriate combination of mono/trivalent cations in high entropy perovskite oxides, could be the effective approach to develop high-performance relaxor ferroelectrics with the desired properties.

## Introduction

The trend in energy storage and conversion technologies has recently evolved. More renewable energy sources, such as solar, wind, and geothermal, have been utilized to replace the use of fossil fuels, which contribute to global warming and air pollution. For optimal electrical generation, a highly efficient and reliable electrical storage system is required. Therefore, it is requirement to have a high energy storage density, an excellent energy storage efficiency, and an ultrafast discharging rate. Dielectric capacitors are an energy storage technology with remarkable qualities, such as high-power density, ultra-fast charge and discharge, and great thermal stability when compared to other devices, such as batteries and electrochemical capacitors. It is typically employed in pulsed powder application, such as electronic circuits, microwave communications, hybrid vehicles, etc. Nevertheless, dielectric capacitors typically have a rather low energy storage density. Therefore, increasing energy storage density is necessary to enhance the performance of dielectric capacitors^1,2^.

Dielectric ceramics have been intensively studied for decades in the field of energy storage capacitors. Among dielectric materials, relaxor ferroelectric ceramics have gained prominence due to their polarization-dependent behavior and dielectric permittivity. Relaxor ferroelectrics are represented by slim polarization–electric field (P-E) loops and a low remnant polarization. Consequently, they display particular characteristics, such as a high dielectric permittivity, a large polarization, high dielectric breakdown strength and low ferroelectric loss which results in high-energy storage performance. In general, energy loss density (W_LOSS_), recoverable energy density (W_REC_), and energy efficiency (η) are critical parameters for determining material energy storage performance. Equations (1)–(3) can be used to calculate these parameters using the P-E hysteresis loop:

1 $${\text{W}}_{\text{LOSS}} = \underset{0}{\overset{{\text{P}}_{\text{MAX}}}{\int }}{\text{EdP}}$$

2 $${\text{W}}_{\text{REC}} = \underset{{\text{P}}_{\text{REM}}}{\overset{{\text{P}}_{\text{MAX}}}{\int }}{\text{EdP}}$$

3 $$\eta = \frac{{{\text{W}}_{{{\text{REC}}}} }}{{{\text{W}}_{{{\text{REC}}}} + {\text{W}}_{{{\text{LOSS}}}} }} \times 100\%$$

where P_MAX_, P_REM_, and E_C_ are maximum polarization, remnant polarization, and coercive field, respectively. Having a high W_REC_, and η indicates that the materials have a good energy storage capacity^1–3^.

The majority of relaxor ferroelectrics are found in complex oxides with ABO_3_ perovskite structures because of cation doping and solid solution formation. This causes the materials to form a system with short-range polar order or polar nanoregions (PNRs) as chemical complexity and compositional heterogeneity increase, resulting in a decrease in hysteresis^4^. Currently, there is increasing interest in a novel oxide system known as high entropy oxides. The concept of the materials is a solid solution with more than or equal to five components in an equimolar proportion which result in a high configurational entropy. Due to the role of high configurational entropy, it is possible to stabilize a single-phase structure^5^. Furthermore, high entropy oxides have intrinsically strong chemical disorder which causes lattice distortion with excellent properties, including hardness, thermal conductivity, dielectric properties, and energy storage performance^6,7^.

Consequently, high entropy oxides have attracted considerable interest in the energy storage capacitor applications. Pu et al.^8^ used a solid-state method to synthesize (Na_0.2_ Bi_0.2_Ba_0.2_Sr_0.2_Ca_0.2_)TiO_3_ high entropy oxides to examine the effect of component disorder on the characteristics of materials. The energy storage properties were discovered for the first time. At 145 kV/cm, it revealed a discharge density of 1.02 J/cm^3^. Later, Wang et al.^7^ synthesized comparable materials using a solid-state technique and investigated their energy storage performance. At a low electric field of 110 kV/cm, an energy storage density of 1.32 J/cm^3^ with a high efficiency of 91% was discovered. To improve energy storage properties, Guo et al.^9^ doped Pb into (Bi_0.2_Na_0.2_Ba_0.2_Sr_0.2_Ca_0.2_)TiO_3_ high entropy oxides to act as a sintering aid and to compensate volatile Bi and Na during preparation in order to increase energy storage performance. It was discovered that Pb can facilitate polarization and hybridization, which is advantageous for enhancing maximum polarization. They have a recoverable energy storage density of 8.2 J/cm^3^ and an ultrahigh efficiency of 92.2% at an ultrafast discharge rate of 5.9 µs and 58.4 kV/mm at ultrahigh voltage.

The alternative synthesis was utilized to enhance the energy storage capabilities of high entropy oxides. Liu et al.^10^ synthesized (Bi_0.2_Na_0.2_ K_0.2_Ba_0.2_Ca_0.2_)TiO_3_ using the flash sintering process to prevent the loss of some components evaporating easily during sintering, such as Na and K. Under an electric field of 129 kV/cm, it was determined that the discharge energy storage density and efficiency of (Bi_0.2_Na_0.2_ K_0.2_Ba_0.2_Ca_0.2_)TiO_3_ high entropy oxides are 0.68 J/cm^3^ and 87.5%, respectively. Yang et al.^11^ synthesized (Bi_0.2_Na_0.2_K_0.2_La_0.2_Sr_0.2_)TiO_3_ using a modified citrate acid technique. The substitution of Sr and La at the A-site in Bi_0.5_Na_0.5_TiO_3_-based (BNT-based) ceramics could disrupt the long-range order of ferroelectric domains, resulting in the formation of polar nano-sized areas that are advantageous to the energy storage capabilities. In addition, it demonstrated a high recoverable energy storage density of 0.95 J/cm^3^ under an electric field of 180 kV/cm. It can be noted that the high-entropy systems have gained popularity and can be used to modify the energy storage properties of electronic ceramics.

In this present work, (A′_0.2_A″_0.2_Ba_0.2_Sr_0.2_Ca_0.2_)TiO_3_ high-entropy perovskite oxides (HEPOs) with different combination of monovalent A-cations, i.e., Na^+^ or K^+^, and trivalent A-cations, i.e., Bi^3+^ or La^3+^, including (Na_0.2_Bi_0.2_Ba_0.2_Sr_0.2_Ca_0.2_)TiO_3_ : NB, (K_0.2_Bi_0.2_Ba_0.2_Sr_0.2_Ca_0.2_)TiO_3_ : KB_,_ (Na_0.2_La_0.2_Ba_0.2_Sr_0.2_Ca_0.2_)TiO_3_ : NL, and (K_0.2_La_0.2_Ba_0.2_Sr_0.2_Ca_0.2_)TiO_3_ : KL, were synthesized using solid-state reaction methods. The effects of mono-/trivalent A-cations of titanate-based high-entropy perovskite oxides on structural, dielectric, ferroelectric, electric, energy storage properties will be investigated.

## Experimental

(A′_0.2_A″_0.2_Ba_0.2_Sr_0.2_Ca_0.2_)TiO_3_ (A′ = Na^+^, K^+^, A″ = Bi^3+^, La^3+^) ceramics was prepared by a solid-state reaction method. Analytical grade reagents of Na_2_CO_3_, K_2_CO_3_, Bi_2_O_3_, La(OH)_3_, BaCO_3_, CaCO_3_, SrCO_3_, and TiO_2_ were used as the raw materials. All starting powders were dried at 100 °C for 24 h to remove moisture. The starting powders were stoichiometrically weighed and mixed by ball milling method in ethanol by using zirconia grinding balls for 24 h. The slurry was dried by vacuum evaporation process with the speed of 90 rpm at 90 °C. The dried powders were calcined in air atmosphere at 975 °C for 2 h with a heating/cooling rate of 5 °C/min in a covered alumina crucible. The 2 wt% polyvinyl alcohol solution was added to the calcined powders by mixing using mortar and pestle until forming dried powders. They were then uniaxially pressed in a cylindrical die under 80 MPa for 2 min to obtain disc pellets or green pellets with 10 mm in diameter and about 1 mm in thickness. The ceramics were sintered at 1250 °C for 2 h with a heating/cooling rate of 2 °C/min in covered alumina crucibles. Finally, the sintered ceramics were polished to less than 0.8 mm in thickness and coated on both sides with a silver paste as electrodes. The coated ceramics were fired at 600 °C for 1 h with the heating/cooling rate of 5 °C/min to eliminate organic content in the silver paste prior to electrical properties measurements.

Rietveld refinement method was carried out using GSAS-II software^12^ to study phase purity and crystallographic structure properties of the ceramics by using of synchrotron X-ray diffraction data collected using Debye–Scherrer geometry with a strip detector (Mythen6K 450 (DECTRIS®) at BL1.1W MXT of the Synchrotron Light Research Institute (SLRI), Thailand. The samples prepared by grinding the ceramic samples into powder and annealed at 400 °C for 1 h were used for synchrotron XRD characterization. Raman spectroscopy measurements (XploRa PLUS, HORIBA) were performed by using a laser wavelength of 532 nm to investigate a short-range structure of the ceramics. X-Ray Fluorescence Spectrometer ((XRF), BRUKER S8 TIGER) was used to determine elemental composition of the ceramics. Shrinkage measurement was performed by measuring linear change in diameter between green pellet and sintered ceramic. Bulk density was determined with Archimedes’ method. Scanning electron microscopy (SEM) were used to investigate surface microstructure of the ceramics (SEM, JEOL JSM-6480LV). Grain size of the ceramics was measured using the linear intercept method. For electrical characterizations, dielectric properties as a function of temperature (25–350 °C) were determined using an LCR-meter (HP model 4192A) at frequencies ranging from 10 to 1000 kHz. Ferroelectric system based on Radiant Precision High Voltage Interface was used to measure the polarization–electric field (P-E) hysteresis loops between 30 °C (Room Temperature, RT) and 150 °C by applying an electric field in the range of 10–100 kV/cm and a frequency of 1 Hz. Similarly, by using the same data from ferroelectric properties measurement, W_REC_, W_LOSS_, and η values were also calculated. The impedance analyzer (Solatron, 1260A) was used to test the complex impedance and complex modulus under frequencies from 0.1 Hz to 1 MHz, and a temperature range from 400 to 500 °C.

## Results and discussion

### Rietveld refinement of synchrotron XRD and Raman Spectroscopy

**Figure 1:**
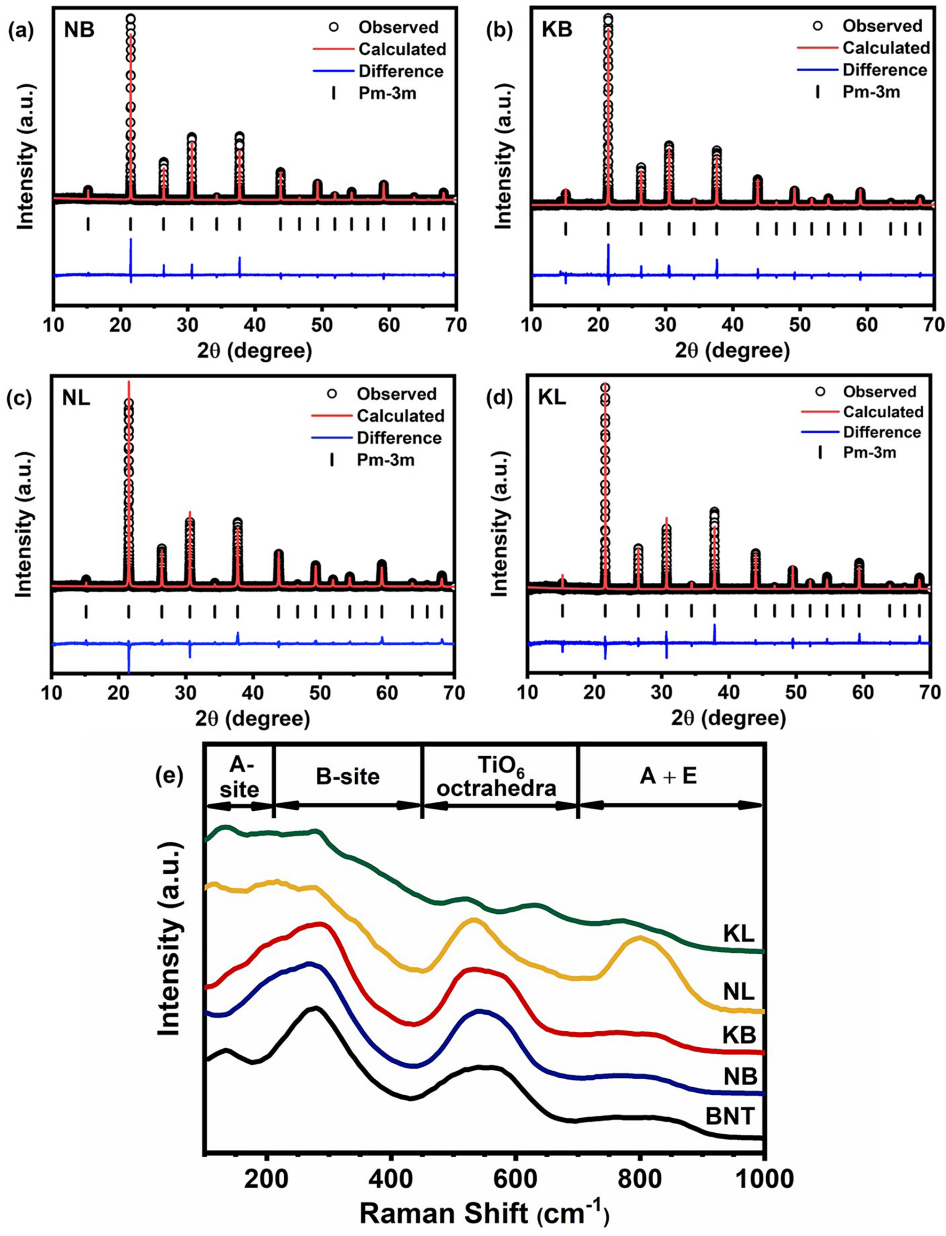
Rietveld refinement analysis of synchrotron X-ray diffraction patterns for (**a**) NB, (**b**) KB, (**c**) NL, and (**d**) KL ceramics (**e**) Raman spectra of NB, KB, NL, and KL ceramics.

Rietveld refinement analysis of synchrotron XRD patterns of NB, KB, NL, and KL ceramics were depicted in Fig. 1a–d. The data including R_w_ (weighted-profile factor), χ^2^, GOF (goodness of fit), crystal structure, lattice parameter, and unit cell volume obtained from refinement were listed in Table 1. The R_w_, χ^2^, and GOF were calculated to evaluate the fitting quality of the experimental data in order to achieve the best possible fit to the experimental diffraction data. All ceramics showed small values of R_w_, χ^2^, and GOF, indicating that refined results agreed with cubic structure (Pm-3 m space group). This suggested that NB, KB, NL, and KL ceramics form an average structure of cubic-perovskite phase. The formation of single-phase perovskite structure with cubic structure agreed well with the calculated Goldschmidt tolerance (t), as shown in Eq. (4)^13^

4 $$\text{t } = \frac{{\bar{\text{R}}_{\text{A}} \, } +{ \, {\text{R}}}_{\text{O}}}{\sqrt{{2} \, }\left({ {\text{R}}}_{\text{B}}+{ {\text{R}}}_{\text{O}}\right)}$$

where ${\bar{\text{R}}}_{\text{A}}$ was defined as the average ionic radii of cation at A-site, including the following: Na^+^ (1.39 Å), K^+^ (1.64 Å), Bi^3+^ (1.36 Å), La^3+^ (1.36 Å), Ba^2+^ (1.61 Å), Ca^2+^ (1.34 Å), and Sr^2+^ (1.44 Å). R_B_ was defined as the ionic radii of cation at B-site, which is Ti^4+^ (0.61 Å). ${\text{R}}_{\text{O}}$ was described as the radii of O^2−^ ion (1.40Å)^14–16^. The calculated tolerance factor for NB, KB, NL, and KL ceramics were 0.995, 1.012, 0.995, and 1.012, respectively. The KB ceramic exhibits larger lattice parameter and unit cell volume than that of NB, NL, and KL ceramics, respectively. It indicated that the different mono-/tri-valent cations at A-site lattice of ceramics affect the structural formation due to ionic size difference of the A-cations.

**Table 1:** The details of refinement parameters.

Composition	Refinement parameters	Structure	Lattice parameter (Å)	Volume (Å^3^)
NB	R_w_ = 11.19% χ^2^ = 3.70 GOF = 1.92	Pm-3 m	a = b = c = 3.91490	60.00
KB	R_w_ = 10.82% χ^2^ = 3.13 GOF = 1.77	Pm-3 m	a = b = c = 3.92636	60.53
NL	R_w_ = 8.43% χ^2^ = 2.22 GOF = 1.49	Pm-3 m	a = b = c = 3.91411	59.67
KL	R_w_ = 7.73 χ^2^ = 1.28 GOF = 1.13	Pm-3 m	a = b = c = 3.90192	59.41

The local structure of NB, KB, NL, and KL ceramics was determined by using Raman spectroscopy by identifying the vibration modes that persisted in the oxide structures. As seen in Fig. 1e, the four distinct bands corresponded to the main Raman modes of perovskite ceramics including 100–200 cm^−1^, 200–450 cm^−1^, 450–700 cm^−1^, and above 700 cm^−1^ were observed for all compositions. The Raman spectrum of the prepared (Bi_0.5_Na_0.5_)TiO_3_ ceramic was included as reference. The first band between 100 and 200 cm^−1^ correlated to the vibration modes of A-site cations (i.e., Na/K–O, Bi/La–O, Ba–O, Ca-O, and Sr–O bonds) in the perovskite structure. The second band in the range of 200–450 cm^−1^ correlated with B-site vibrations (Ti–O bonds). The third band of 450–700 cm^−1^ related to the stretching vibrations of TiO_6_ octahedral. The A and E overlapping bands in the final region above 700 cm^−1^ related with oxygen displacements. The NB and KB spectra displayed distinct characteristics between 100 and 200 cm^−1^ than that of the NL and KL spectra. It could be attributed to different cation disorder at A-site caused by Bi and La ion resulting in different bonding characteristics of A-O bonds. Moreover, according to literature, the presence of hybridization of 6s^2^ bismuth lone pair and oxygen p-orbital played a vital part in local structure of bismuth sodium titanate perovskites^17^. The weaker A-O bond could be induced by introducing La onto A-site and by increasing temperature to depolarizing temperature, suggesting loss of the hybridization of 6s^2^ bismuth lone pair and oxygen p-orbital^17–19^. A clear observation in distinct A-O bands between NB/KB and NL/KL suggested that the presence or the lack of hybridization of 6s^2^ bismuth lone pair and oxygen p-orbital was crucial for short range structure formation. It affected macroscopic electrical properties including dielectric permittivity and ferroelectric behaviors, as mentioned later. The different characteristics observed within the band between 200–450 cm^−1^ and 450–700 cm^−1^ are likely associated with the change in local structure due to the presence of different A-cation combinations^18^. This indicated that local Ti–O bond and TiO_6_ octahedral vibration of the NB, KB, NL, and KL ceramics was affected by the composition of the A site, particularly mono-/trivalent cations. The characteristic of the cubic phase, which is identified by a low intensity peak at around 800 cm^−1^, was observed in NB, KB, and KL ceramics. Nonetheless, the NL peak exhibits an increase in intensity, confirming the transition from cubic to tetragonal phase^20–24^. It should be noted that the overall Raman spectra of bismuth containing compounds (NB and KB) exhibited similar peak shape and modes to BNT ceramic while the NL and the KL revealed strong deviation from the BNT ceramic.

### Linear shrinkage, density, and relative density measurement

**Figure 2:**
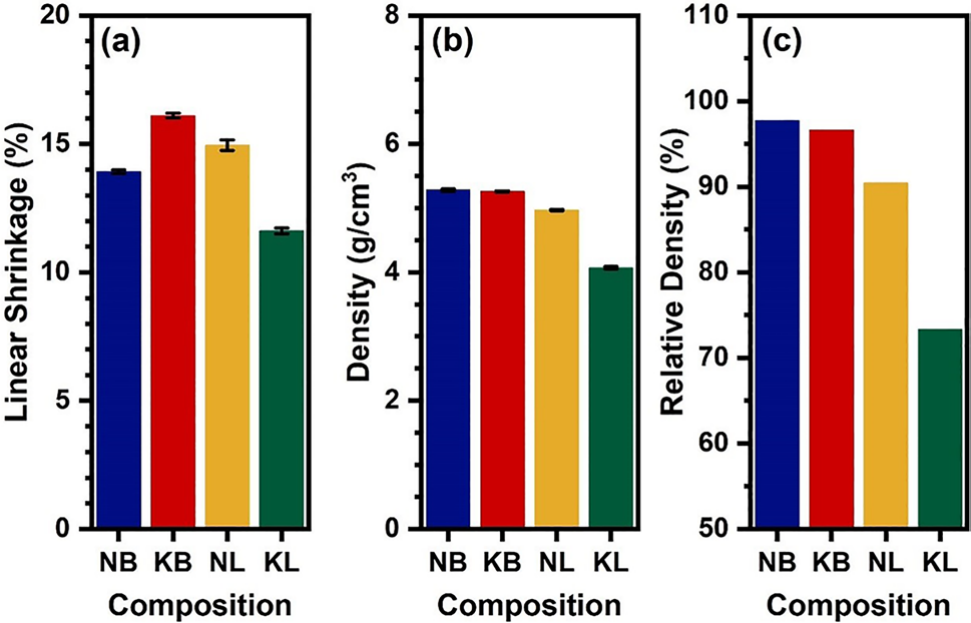
Change in (**a**) linear shrinkage (%) and (**b**) density and (**c**) relative density (%) of NB, KB, NL, and KL ceramics.

As shown in Fig. 2a–c, the linear shrinkage, density, and relative density of the sintered NB, KB, NL, and KL ceramics were evaluated to determine the effect of various mono- and tri-valent A cation substitutions on densification. The KB ceramic showed the largest linear shrinkage (16.1%). Meanwhile, the linear shrinkages of NL and NB ceramics were lower with values of 14.91% and 13.92%, respectively. The KL ceramic exhibited the lowest linear shrinkage at 11.61%. Under same conditions, the NB and KB ceramics attained high densities of 5.28 g/cm^3^ and 5.26 g/cm^3^, respectively. The NB ceramic showed a slightly higher relative density than KB ceramic with values of 97.72% and 96.61%, respectively. The relative density value of 95% or more was expected for dense ceramics. This lower value was an indication of greater porosity^25^. In comparison, the NL and KL ceramics persisted lower densities with values of 4.77 g/cm^3^ and 4.07 g/cm^3^, respectively. Both NL and KL ceramics were highly porous with the relative densities of 90.43% and 73.33%, respectively. This demonstrates that the ceramic was dense with compositions containing the Bi^3+^ ion, as compared to the La^3+^ ion. Since La_2_O_3_ exhibited a melting point (2315 °C) which is significantly higher than that of Bi_2_O_3_ (817 °C). Compositions containing lower melting temperature components were able to densify at lower temperatures^26–28^. It should be noted that higher sintering temperature, i.e., 1275 °C, for the KL and NL samples were carried out. However, the obtain ceramics, which showed similar densities and shrinkage, exhibited partially melt surface and warping. Therefore, it was not suitable for further investigation of these samples.

### Scanning electron microscope (SEM) microstructure analysis

**Figure 3:**
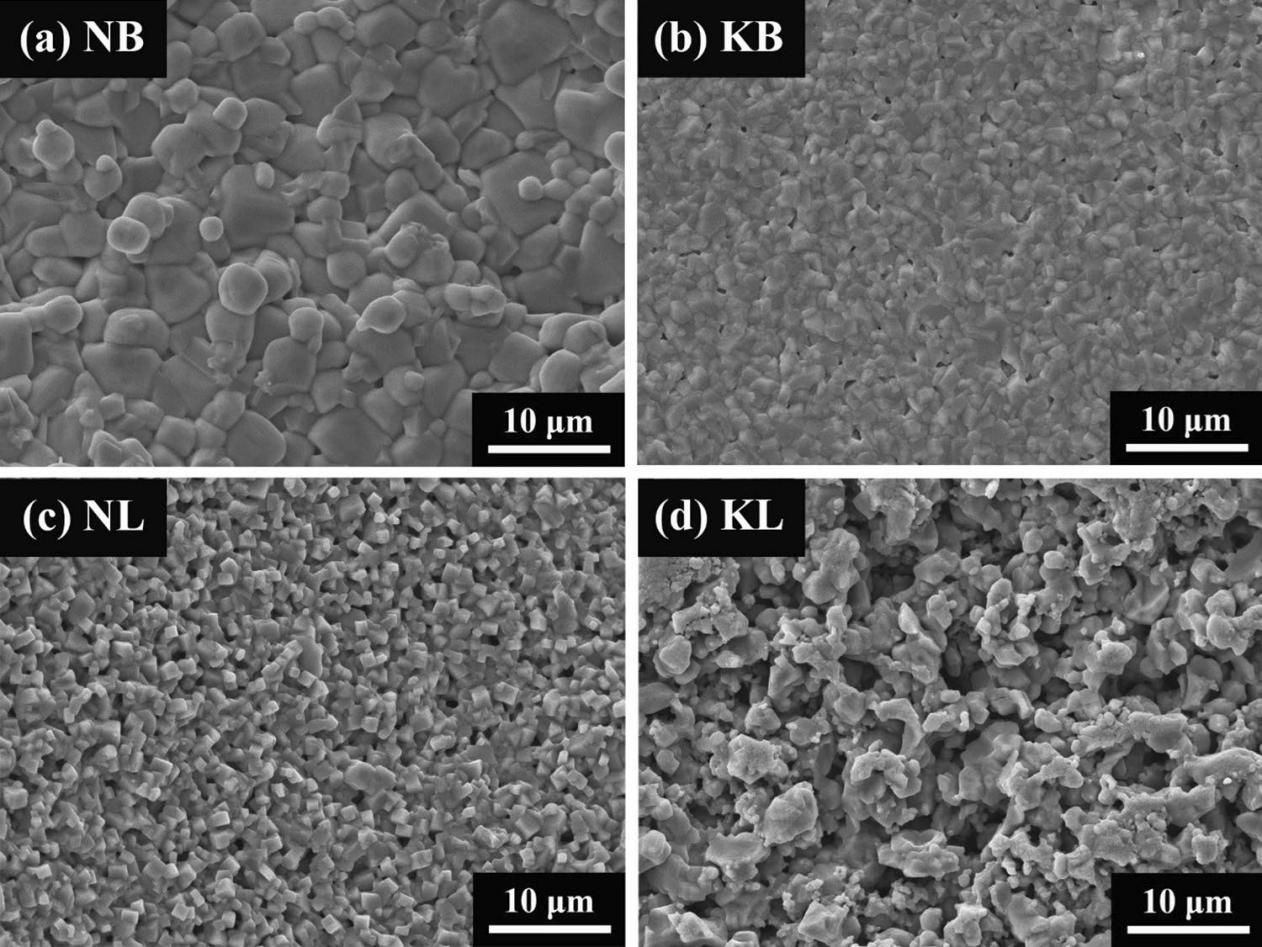
SEM images of (**a**) NB, (**b**) KB, (**c**) NL, and (**d**) KL ceramics.

Microstructure of NB, KB, NL, and KL ceramics sintered at 1250 °C were evaluated. As shown in Fig. 3a–d, the grain structure of NB ceramic was highly dense. The grain size as measured was 3.10 μm. The KB ceramic exhibited a dense and smaller grain size of 1.34 μm. The NL ceramic featured cubic-shaped grains. It exhibited grain size of 1.39 μm and showed visible porosity. The KL ceramic exhibited agglomerated particles with large pore sizes. All ceramics corresponded to the relative density values as illustrated in Fig. 2c. The grains in the NB and KB ceramics were larger and denser than those in the NL and KL ceramics. This could be due to the influence of Bi^3+^ , which rapid diffusion coefficient of Bi^3+^ ion allowed diffusion to occur more efficiently, thus grain growth was expected^26^. Meanwhile, the addition of La^3+^ resulted in a reduced sintering diffusion rate, resulting in finer grains. It had been observed that the La^3+^ ion could reduce the resultant grain size at low doping concentrations (≤ 0.2 at.%) by inhibiting grains growth^29–31^.

Furthermore, the estimated elemental compositions of the ceramics were determined using XRF analysis. Table 2 lists the weight and atomic percentages of the elements, excluding oxygen, found in NB, KB, NL, and KL ceramics. The ideal atomic percentages should consist of 4 at% of each element on the A-site and 20 at% of Ti for compositions containing five equimolar elements on the A-site. The results indicate that atomic percentages of the constituent elements were estimated to be relatively close to their expected values from the batch calculations. It should be noted that the errors obtained were found to be less than 1.00% for Ti and Sr, and between 1.00 and 2.00% for other elements except light element. The higher error values of Na and K were from weak fluorescence signals.

**Table 2:** XRF analysis of elemental compositions in the NB, KB, NL, and KL ceramics.

Composition	Element	Weight concentration (%)	Atomic concentration (%)	Statistical *error* (%)
NB	Na	2.75	4.62	4.40
	Bi	21.10	3.90	0.39
	Ba	14.50	4.08	1.74
	Ca	4.43	4.27	1.74
	Sr	7.72	3.41	0.37
	Ti	24.8	20.03	0.74
KB	K	3.09	3.10	2.21
	Bi	20.50	3.85	0.41
	Ba	14.50	4.14	1.81
	Ca	4.24	4.15	1.86
	Sr	7.51	3.36	0.38
	Ti	25.50	20.91	0.76
NL	Na	3.39	5.29	3.89
	La	15.9	4.10	1.31
	Ba	15.30	3.99	1.38
	Ca	4.47	4.00	1.32
	Sr	7.94	3.25	0.29
	Ti	26.40	19.77	0.58
KL	K	3.62	3.44	1.66
	La	16.20	4.34	1.45
	Ba	15.4	4.17	1.53
	Ca	4.20	3.90	1.55
	Sr	8.64	3.67	0.30
	Ti	26.00	20.20	0.65

### Dielectric properties

**Figure 4:**
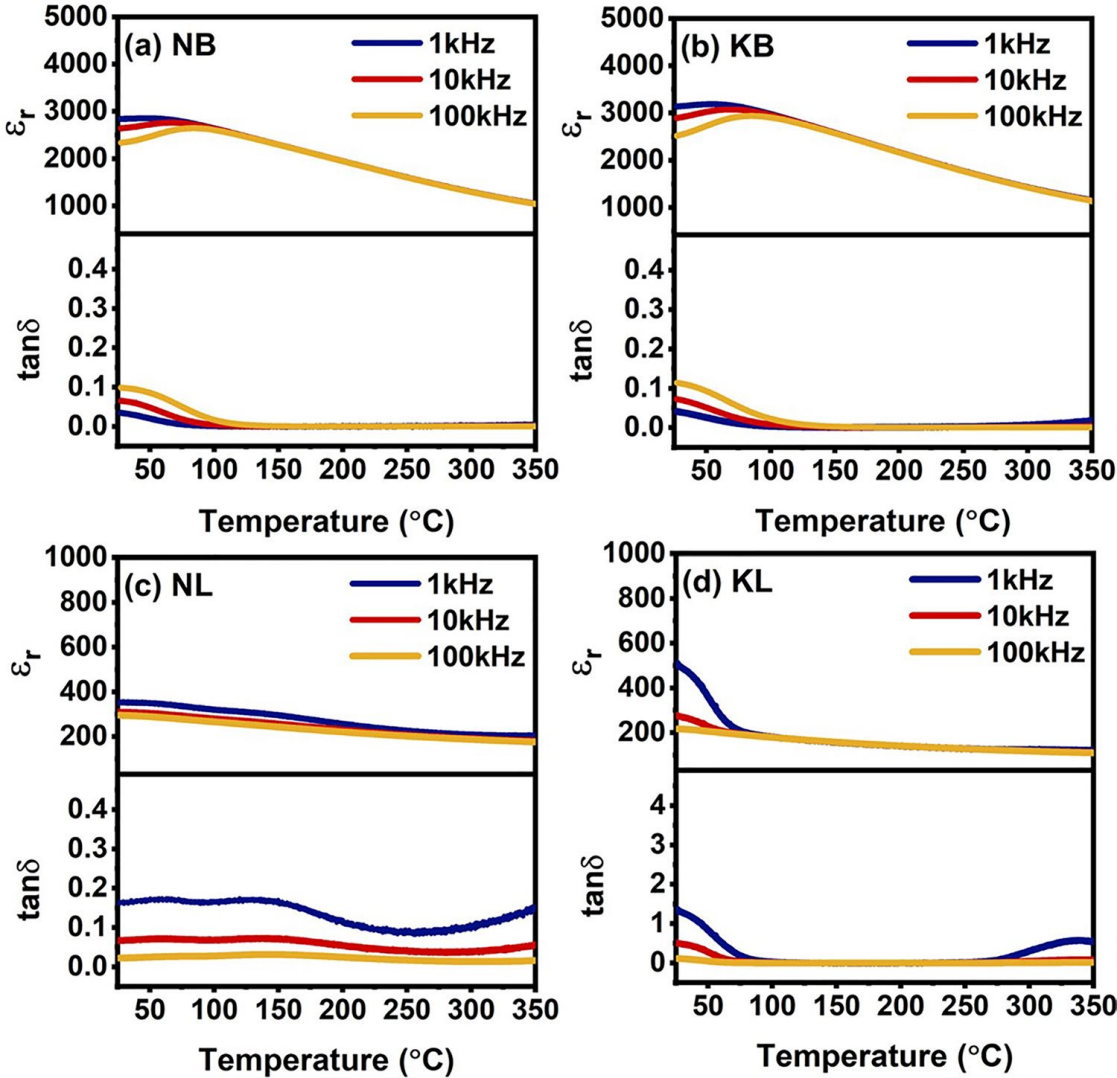
Temperature dependent dielectric responses at various frequencies of (**a**) NB, (**b**) KB, (**c**) NL, and (**d**) KL ceramics.

The temperature dependence of the dielectric response was measured over the frequency range of 1 to 100 kHz and over the temperature range from room temperature to 350 °C. The relative permittivity and dielectric loss of the NB ceramics were shown in Fig. 4a. The maximum relative permittivity and dielectric loss were seen near room temperature and became broad towards higher temperatures. The relative permittivity tended to decrease with increased temperature, whereas the dielectric loss was relatively low and increased with increased temperature. It could be noted that the NB ceramic exhibited relaxor ferroelectric behavior from its dielectric response. The relative permittivity and dielectric losses of the NB and KB ceramics were relatively similar values, 3000 and 0.15, respectively at room temperature. The temperature dependence of relative permittivity and dielectric loss of KB ceramic was displayed in Fig. 4b. A high relative permittivity peak gradually transformed to a broad peak over the wide temperature range. The similar frequency phenomenon appeared with the NB ceramic. This was an indicative of a relaxor ferroelectric behavior. Figure 4c revealed that the relative permittivity and dielectric loss of the NL ceramic decreased with increasing frequencies. The relative permittivity was about 400 to 200 from room temperature to 350 °C with dielectric loss of less than 0.2 over a wide range temperature. The drastic reduction in the relative permittivity as a function of temperature caused by the increase of degree of disorder of dipoles^32^. However, at high temperatures, from 250 °C to 350 °C, the dielectric loss increased gradually at higher temperature, suggesting the onset of conduction. This phenomenon revealed that relaxor ferroelectric behavior was not observed for the NL ceramic. Figure 4d showed a strong frequency dependence of relative permittivity and dielectric loss of the KL ceramic at the temperature of below 100 °C. At higher temperature, the relative permittivity and dielectric loss of the KL ceramic with various frequencies remained stable. However, there was an increase in dielectric loss of the KL ceramic at high temperature due to conductive characteristics.

**Figure 5:**
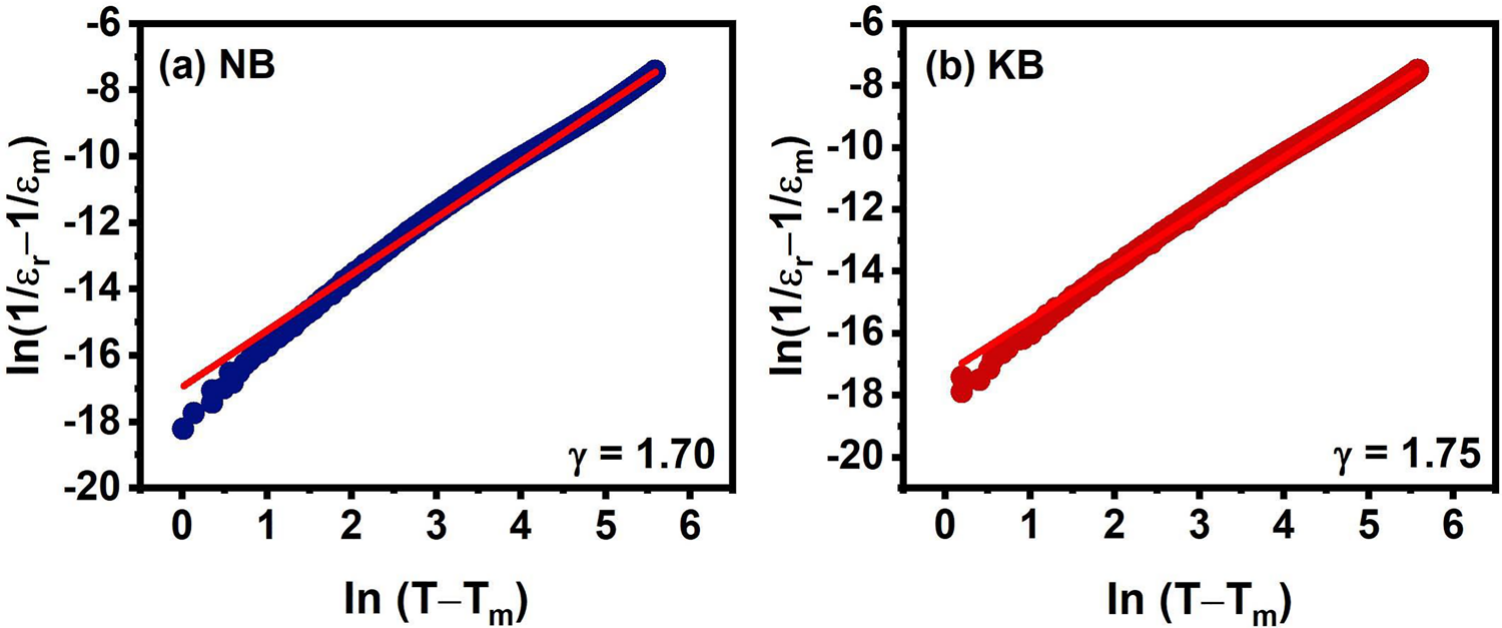
Relationship between ln(1/ε_r_ − 1/ε_m_) and ln(T − T_m_) measured at 100 kHz of (**a**) NB and (**b**) KB ceramics. The red solid lines are the fit to the Modified Curie–Weiss law.

To quantitatively characterize the relaxor behavior of the ceramics, the modified Curie–Weiss law was used to calculate the degree of relaxation characteristic or the degree of diffuseness (γ) which could be expressed by the following equations:

5 $$\frac{1}{{\varepsilon_{{\text{r}}} }} - \frac{1}{{\varepsilon_{{\text{m}}} }} = \frac{{({\text{T}} - {\text{T}}_{{\text{m}}} )^{\gamma } }}{{\text{C}}}$$

where γ represented the dielectric relaxation factor which lie between 1 and 2, and ε_m_ represented the maximum dielectric constant. The value of γ equal to 1 represents a normal ferroelectric transition whereas a γ value equal to 2 represents a relaxor ferroelectric transition^33^. The relationship between ln(1/ε_r_ − 1/ε_m_) and ln(T − T_m_) of the ceramics was displayed in Fig. 5a,b. The γ value for the NB and KB ceramics was calculated to be 1.70 and 1.75, respectively. These values were in a similar range of (1.46–1.85) as found in other titanate-based relaxor ferroelectrics^7,9,20,34^. The KB ceramic was found to have the maximum γ and exhibited a high dielectric permittivity of over 3000 at 1 kHz over a wide temperature range due to the presence of the relaxor state^35^. It should be noted that the dielectric characteristics of KL and NL did not show relaxor ferroelectric characteristics, i.e., dielectric peaks occurred at different frequencies. Therefore, the degree of diffuseness of the KL and NL sample was not analyzed.

### Ferroelectric behavior and energy storage properties

**Figure 6:**
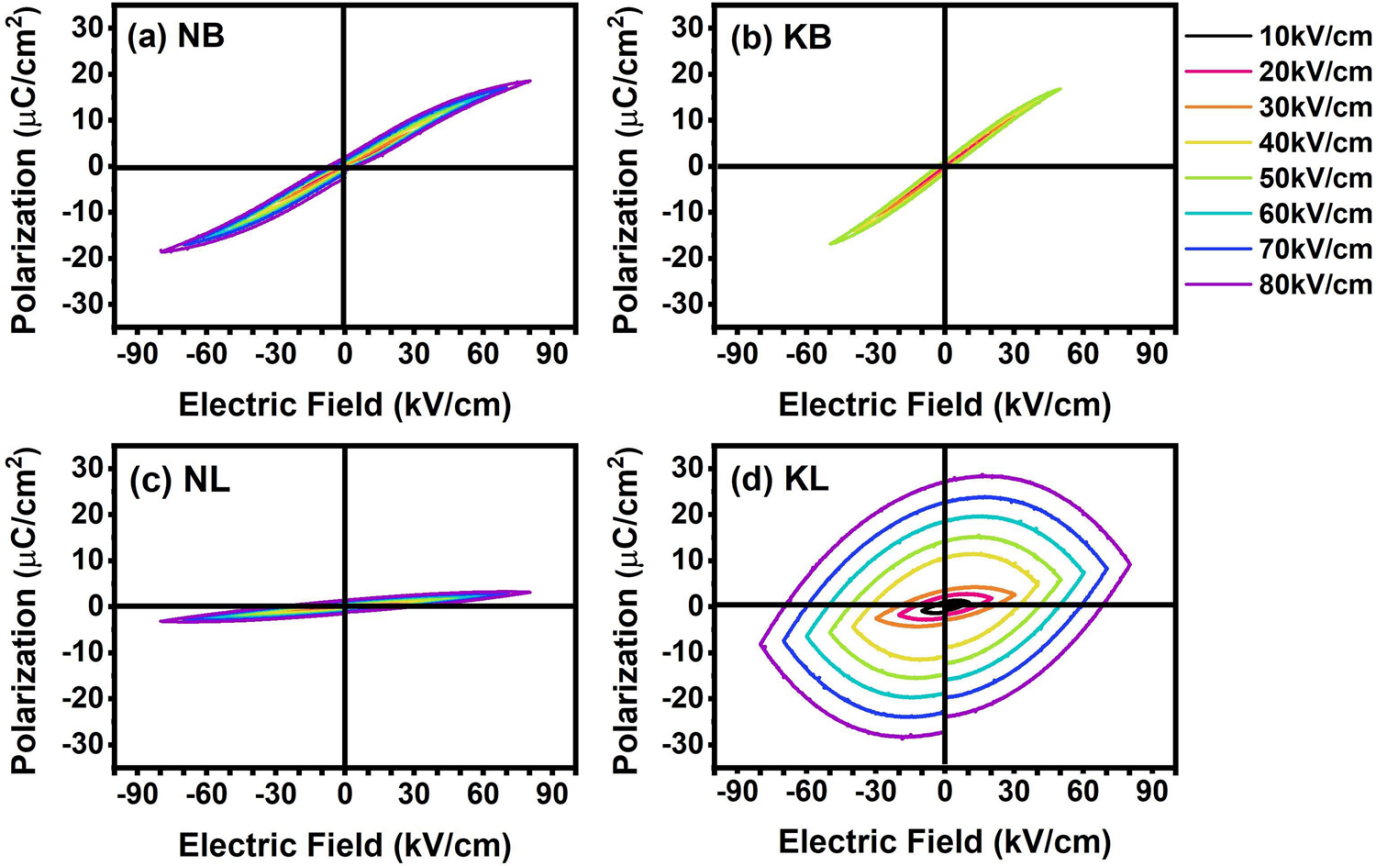
Polarization–Electric field (P-E) hysteresis loops at different applied electric field ranging from 10 kV/cm to 80 kV/cm of (**a**) NB, (**b**) KB, (**c**) NL, and (**d**) KL ceramics.

As shown in Fig. 6a–d, the room-temperature P-E hysteresis loops under different applied electric fields at 1 Hz were demonstrated. It could be seen that the slim loops were observed in NB, KB, and NL ceramics. However, the rounded loops were exhibited in the KL ceramic, suggesting high loss behavior under high electric field. The NB, NL, and KL ceramics reached breakdown electric fields of around 80 kV/cm, whereas the KB ceramic could withstand only 50 kV/cm. The slim characteristics of the P-E loops is indicative of relaxor ferroelectric nature for the NB and KB ceramics. The NL ceramic showed linear dielectric behavior. The KL ceramic showed lossy dielectric behavior.

**Figure 7:**
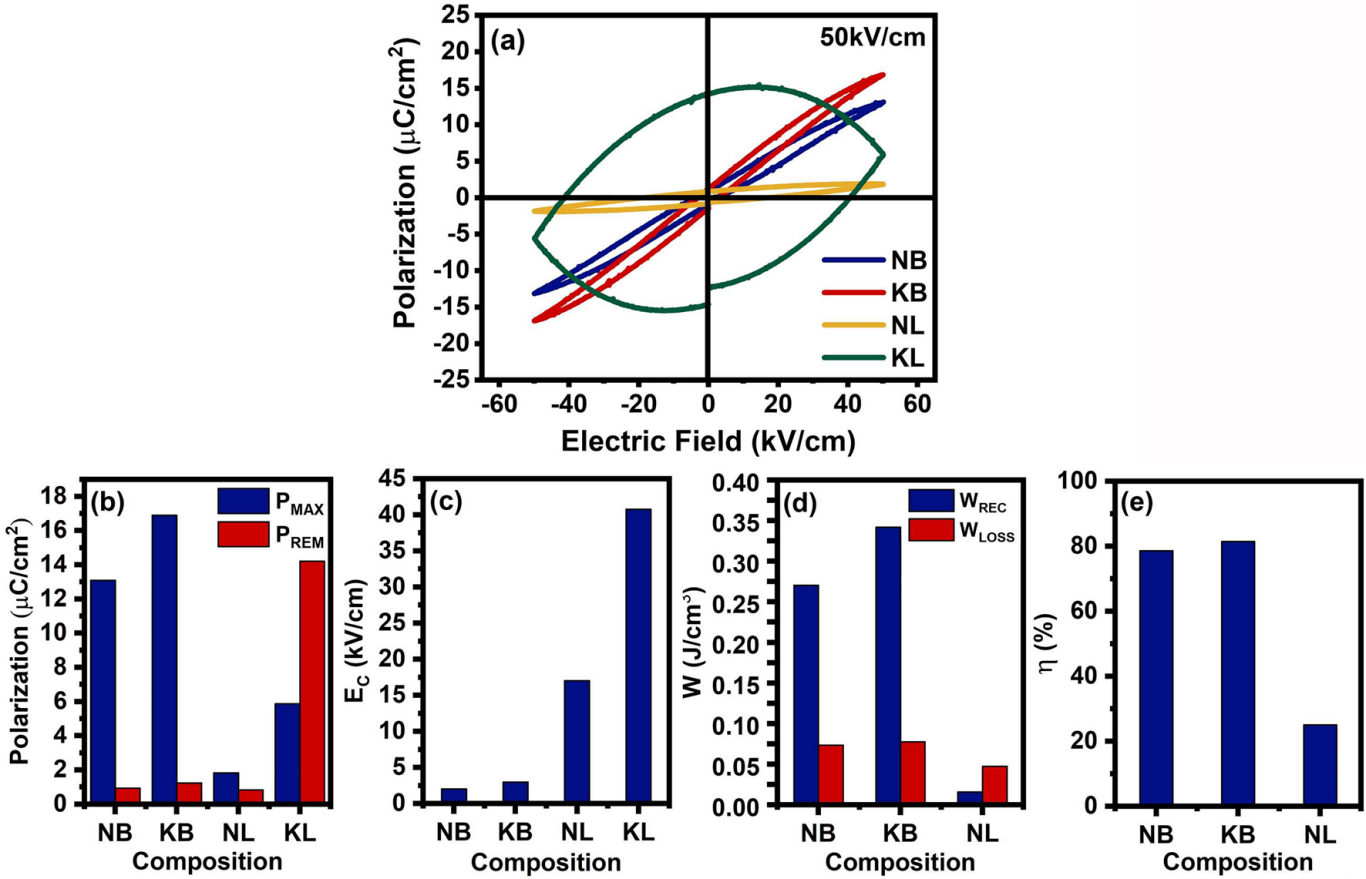
(**a**) P-E hysteresis loops, (**b**) relation between P_MAX_, P_REM_ and composition, (**c**) relation between E_C_ and composition, (**d**) relation between W_REC_, W_LOSS_ and composition, and (**e**) relation between ŋ and composition of NB, KB, NL, and KL ceramics at 50 kV/cm.

The P-E loops measured at 50 kV/cm of ceramics were selected for comparison, and their corresponding electrical properties were presented in Fig. 7a–c. As for slim loops, the KB ceramic clearly displayed the greatest P_MAX_, followed by the NB and NL ceramics. All ceramics exhibited noticeable remnant polarization (P_REM_) values that were not drastically different. The highest P_MAX_ of 16.9 μC/cm^2^ with P_REM_ of 1.23 μC/cm^2^ were achieved in the KB ceramic. The NB ceramic exhibited P_MAX_ of 13.1 μC/cm^2^ with P_REM_ of 0.93 μC/cm^2^. The NL ceramic showed P_MAX_ of 1.83 μC/cm^2^ with P_REM_ of 0.84 μC/cm^2^. Coercive filed (E_C_) values for all ceramics were in the range of 2.05 to 2.98 kV/cm for NB and KB ceramics. According to the concept of high entropy oxides, reduction of P_REM_ resulted from random occupancy of multiple ions at the A-site, which disrupt polar ordering. Therefore, the NB and KB ceramics showed slim P-E loops with high P_MAX_ and low P_REM_ values, indicating relaxor behavior. The NL ceramic was a linear dielectric due to its linear response and lower dielectric permittivity.

The corresponding energy storage properties were calculated and plotted in Fig. 7d,e. Maximum recoverable energy density (W_REC_) of 0.34 J/cm^3^ was achieved in KB ceramic when applied to electric field of 50 kV/cm. The NB ceramic exhibited W_REC_ of 0.27 (0.52 J/cm^3^ at 80 kV/cm), whereas the NL ceramic showed W_REC_ of 0.01 J/cm^3^. All ceramics exhibited similar energy loss density (W_LOSS_) values. W_LOSS_ for KB ceramic was 0.08 J/cm^3^, W_LOSS_ for NB ceramic was 0.08 J/cm^3^ (0.23 J/cm^3^ at 80 kV/cm), and W_LOSS_ for NL ceramic was 0.05 J/cm^3^. Consequently, the greatest energy efficiency (ŋ) was 81.51%, achieved by the KB ceramic. Lower value for NB ceramic was 78.65% (69.76% at 80 kV/cm), whereas those for NL ceramic was 24.99%. Higher energy storage density benefited from the relaxor behavior of KB and NB ceramics. Thus, it was more effective in storing energy due to the low energy barrier of nanodomains (PNRs). The energy storage density of the ceramics followed similar trends as other high entropy perovskite oxides based on titanate, such as (Na_0.2_Bi_0.2_Ba_0.2_Sr_0.2_Ca_0.2_)TiO_3_ (1.02 J/cm^3^ at 145 kV/cm)^8^, (Bi_0.2_Na_0.2_Ca_0.2_Sr_0.2_Ba_0.2_)TiO_3_ (1.32 J/cm^3^ at 110 kV/cm)^7^, (Bi_0.2_Na_0.2_Ba_0.2_Sr_0.2_Ca_0.2_)TiO_3_ (1.3 J/cm^3^ at 150 kV/cm)^9^, (Ba_0.2_Na_0.2_K_0.2_La_0.2_Bi_0.2_)TiO_3_ (1.062 J/cm^3^ at 100 kV/cm)^36^, and (Bi_0.2_Na_0.2_K_0.2_Ba_0.2_ Ca_0.2_)TiO_3_ (0.684 J/cm^3^ at 129 kV/cm)^10^. Therefore, the KB ceramic was a promising candidate for using as energy storage devices.

### Temperature-dependent ferroelectric behavior

**Figure 8:**
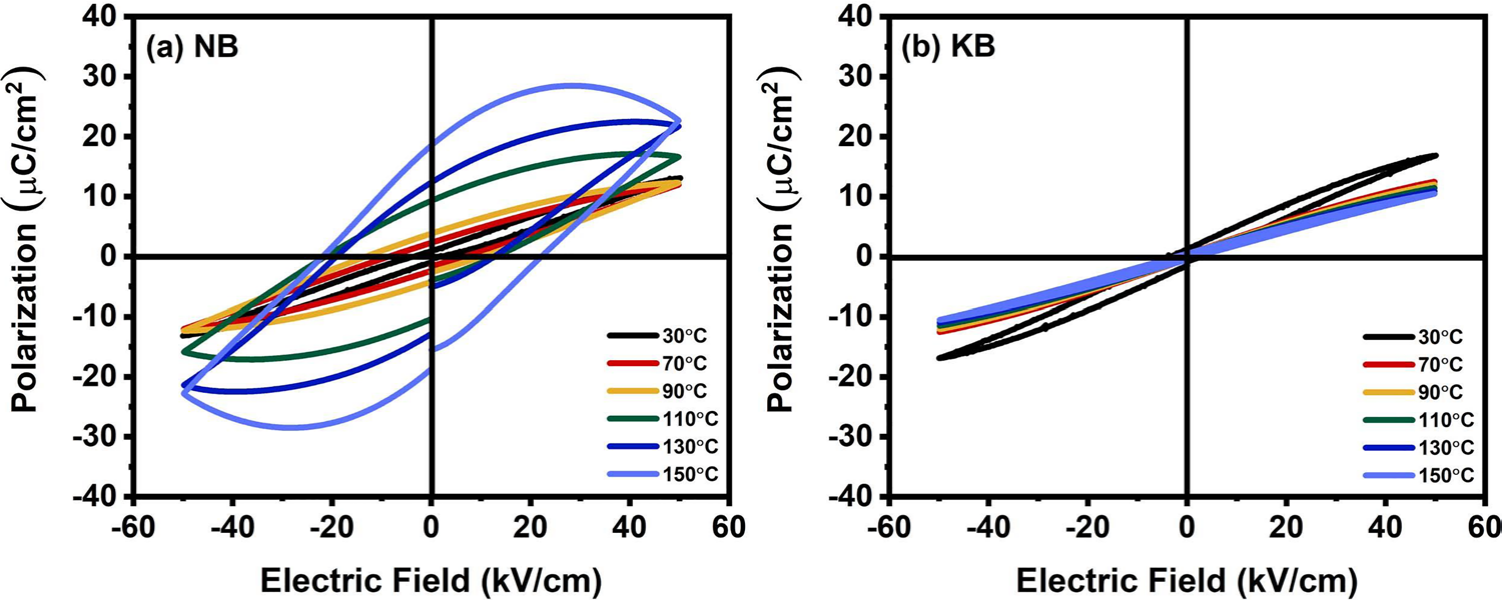
Temperature dependence on P-E hysteresis loops of (**a**) NB and (**b**) KB ceramics measured under an electric field of 50 kV/cm and a frequency of 1 Hz.

Figure 8a,b showed the temperature dependence of P-E hysteresis loops of NB and KB ceramics at 50 kV/cm, respectively. At 30 °C (room temperature), the P-E loops of the NB ceramics displayed a relaxor ferroelectric behavior, and at temperatures between 30 °C and 90 °C, the P_REM_ values tended to rise, which could be due to the increase of leakage current leading to increasing polarization current for relaxor ferroelectric^37^. When the temperature exceeded 100 °C, the P-E loops clearly exhibited a higher loss tendency. The P-E loops of KB ceramics showed the relaxor ferroelectric behavior at 30 °C. The P-E loops displayed a slim characteristic with decreased P_REM_, and remained relaxor ferroelectric as the temperature increased. This suggests diffuse phase transition behavior, which was typical of a relaxor ferroelectric material. The decrease in polarization exhibited a strong ergodicity^38^. Thus, the ferroelectric behavior of NB and KB ceramics was affected by the difference in monovalent cations and temperature.

### Impedance and electric modulus measurement

**Figure 9:**
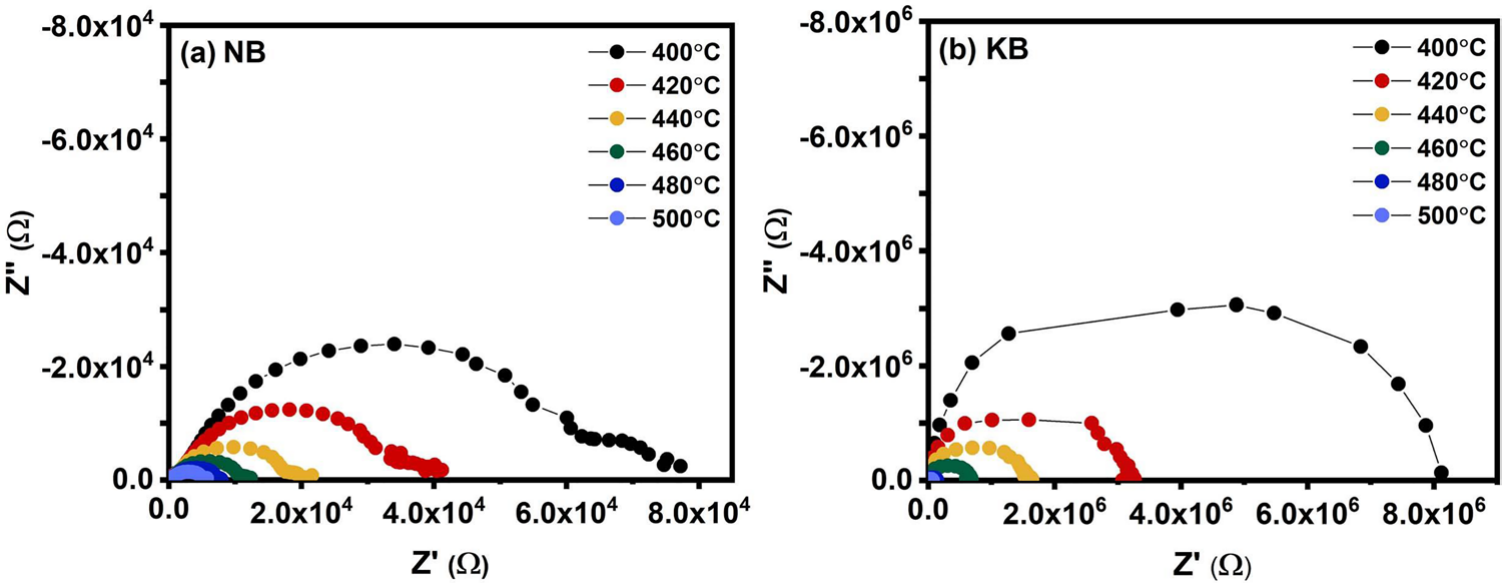
Complex impedance plot between the imaginary part (Z″) and the real part (Z′) of (**a**) NB and (**b**) KB ceramics in the temperature range of 400 °C–500 °C.

The complex impedance and electric modulus measurements were conducted for the purpose of investigating the conduction mechanisms in these materials, with the goal of differentiating between bulk and grain boundary contributions to the relaxation phenomena. The temperature dependence of the complex impedance of NB and KB ceramics over the temperature range 400 °C–500 °C is shown in Fig. 9. At the measured temperatures, the data for the NB ceramic in Fig. 9a showed two semicircles and tended to decrease as the temperature increased. These features were attributed to the effects of grain (high frequency) and grain boundary (low frequency) contributions to the impedance. This could likely be due to the inhomogeneous distribution defects associated with volatile cations in the ceramic^39^. In contrast, the KB ceramic in Fig. 9b displayed a single semicircle and decreased as the temperature increased, indicating a decrease in the DC resistivity^40^.

**Figure 10:**
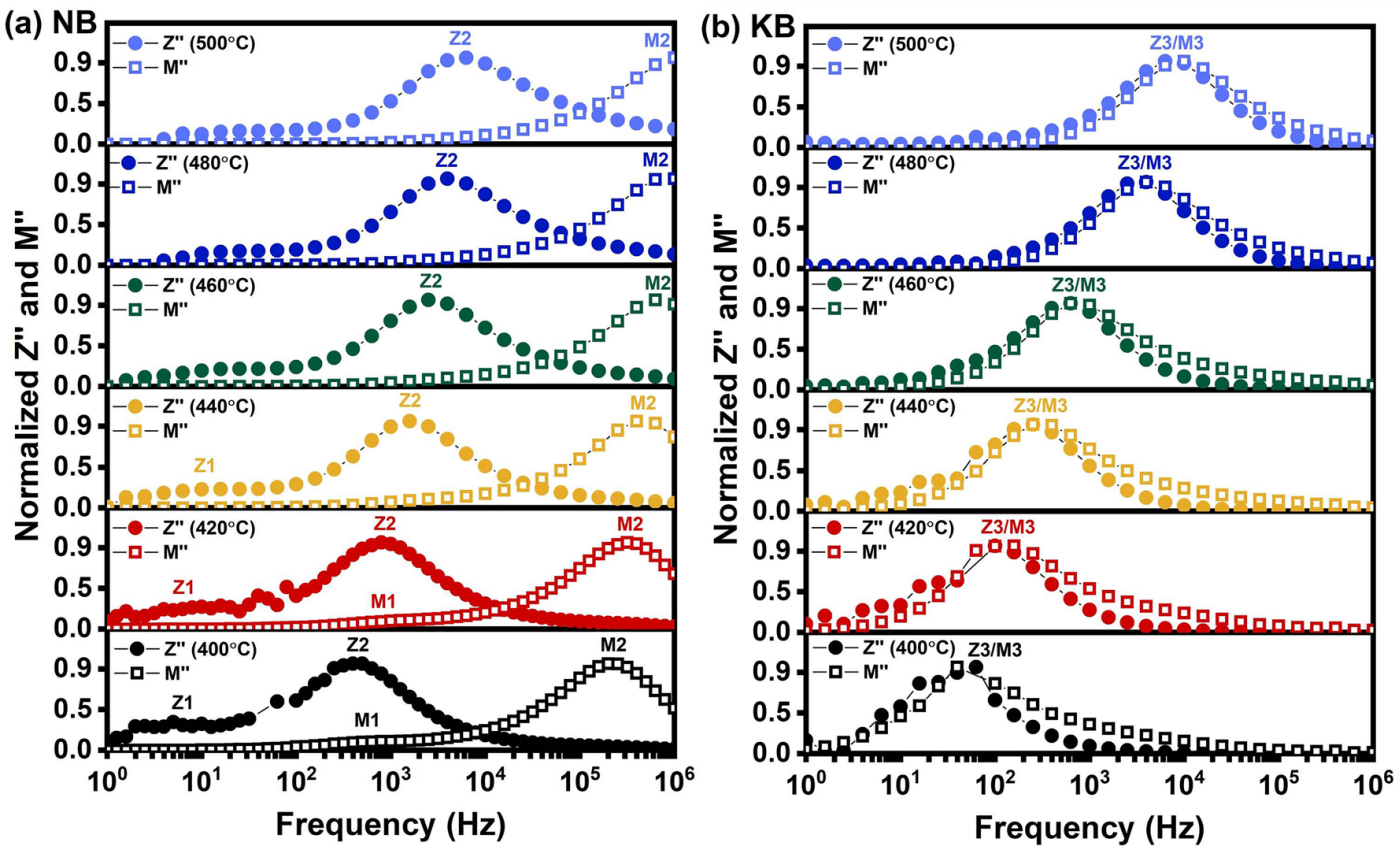
Frequency dependence of the imaginary part of impedance (Z″) and electrical modulus of (a) NB and (b) KB ceramics in the temperature range of 400 °C–500 °C.

Detailed analysis on the trend of Z′′ and M′′ values for NB and KB ceramics as a function of frequency over the temperature range of 400 °C–500 °C was further analyzed to identify conduction mechanisms and relaxation process as shown in Fig. 10. The semi-log plots of normalized Z′′ and normalized M′′ as a function of frequency showed multiple peaks that relate to the transport and relaxation processes^40^. In Fig. 10a, the NB ceramic exhibited two peaks of normalized Z′′. The first peak (Z1) at lower frequencies was poorly resolved and largely disappeared as the temperature increased. A major peak (Z2) was apparent at all temperatures and clearly shifted to higher frequencies as the temperature increased. In the modulus data for NB at low temperatures, there is evidence of a small peak (M1) which was correlated to the main impedance peak (Z2). A much larger peak in modulus (M2) was apparent at all temperatures and was not correlated to the impedance peaks Z1 or Z2. This is indicative of an electrical heterogeneity in the microstructure which can most likely be attributed to differences in the stoichiometry of grain and grain boundary regions. In contrast, the impedance and modulus data for the KB ceramic showed close correlation with a large peak in impedance and modulus (Z3/M3) appearing at the same frequency at all temperatures. This is indicative of a more electrically homogeneous microstructure.

**Figure 11:**
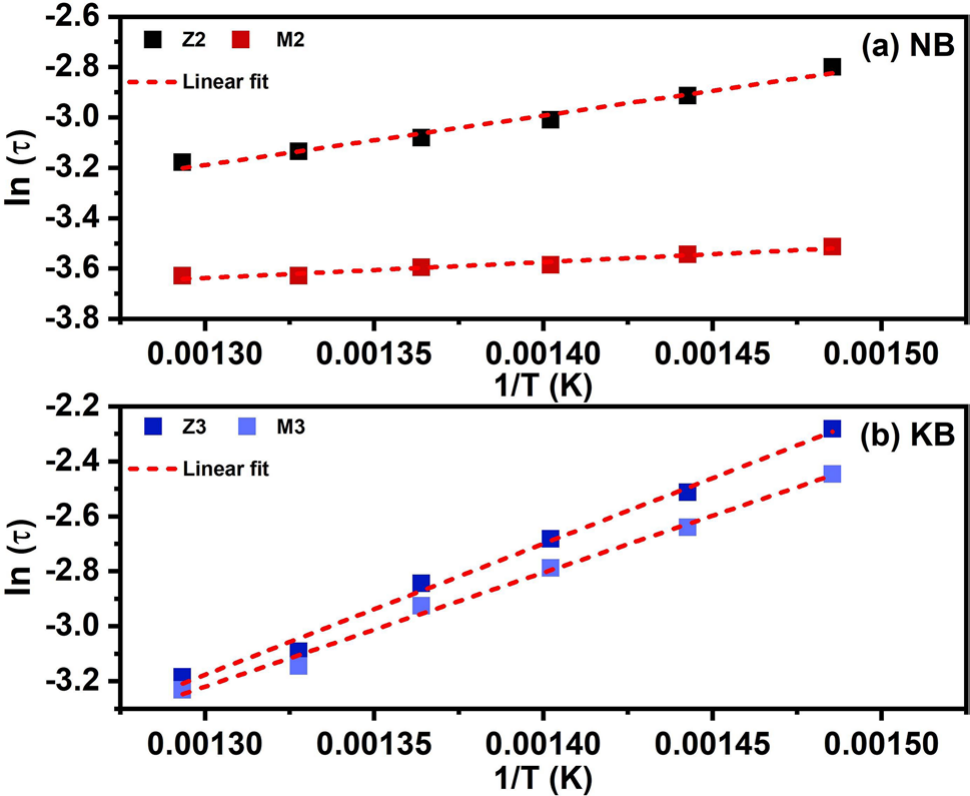
Arrhenius plot of relaxation time (τ) for (**a**) NB and (**b**) KB ceramics in the temperature range of 400 °C–500 °C.

The variation of relaxation time (τ) as a function of temperature for NB and KB ceramics derived from the impedance data were shown in Fig. 11a,b, respectively. The τ value of both ceramics followed the Arrhenius relationship as presented in the Eq. (6) where τ_o_ is pre-exponential factor, k_B_ is Boltzman constant, T is the absolute temperature, and E_a_ is activation energy.

6 $$\tau = \tau o\,\exp ( - {\text{E}}_{{\text{a}}} /{\text{k}}_{{\text{B}}} {\text{T}})$$

The activation energies obtained from this fitting are listed in Table 3. These data show that the NB ceramic had the higher values of E_a_ in grain boundary than that of the bulk regions. For KB ceramic, the E_a_ values obtained from Z″ and M″ were nearly similar which again is indicative of an electrically homogeneous structure. However, the activation energies in grain boundary and grain of KB ceramic were higher than in NB ceramic. As the Z′′ and M′′ peaks represented dielectric relaxation (localized conduction) and long-range conduction (non-localized conduction), respectively^41^. In other words, for NB ceramics, the separation of peak frequencies between Z′′ and M′′ indicated that the domination of relaxation process by localized conduction of charge carriers. On the other hand, the non-mismatch of peak frequencies between Z′′ and M′′ of KB ceramics suggested the non-localized conduction process of charge carriers.

**Table 3:** Parameters obtained from fitting relaxation time (τ) of imaginary part of impedance (Z″) and Imaginary part of electric Modulus (M″).

Composition	E_a_ (eV)			
	Z″		M″	
	Z2	Z3	M2	M3
NB	0.17	–	0.05	–
KB	–	0.41	–	0.36

Overall, the selected titanate perovskites based on equimolar five-component A-cations could be prepared by conventional solid state reaction method. It was clear that monovalent cations (Na^+^ or K^+^) show slight effects on average and local structure formation as well as dielectric properties. More importantly, the compound consisting distinct trivalent cations, i.e., Bi^3+^ or La^3+^, resulted in different local structure formation, dielectric properties, and ferroelectric behavior. This is likely due to the presence of the Bi^3+^ ion with lone-pair electronic structure which drives structural distortions^42^. Thus, the polar structure of the nano scale (i.e. PNRs) could be maintained in the NB and KB ceramics.

## Conclusion

High-entropy perovskite oxides composed of (A′_0.2_A″_0.2_Ba_0.2_Sr_0.2_Ca_0.2_)TiO_3_ (A′ = Na^+^, K^+^, A″ = Bi^3+^, La^3+^) were synthesized using a conventional solid-state reaction. The average grain sizes ranged between 1.34 µm to 3.10 µm and the (Na_0.2_Bi_0.2_Ba_0.2_Sr_0.2_Ca_0.2_)TiO_3_ and (K_0.2_Bi_0.2_Ba_0.2_Sr_0.2_Ca_0.2_)TiO_3_ ceramics exhibited high relative density of more than 95%. These compounds had the highest dielectric constant and the lowest dielectric loss of ~ 3000 and ~ 0.1 at 1 kHz, respectively. The (K_0.2_Bi_0.2_Ba_0.2_Sr_0.2_Ca_0.2_)TiO_3_ ceramic exhibited the highest maximum polarization and lowest remnant polarization, corresponding to relaxor ferroelectric behavior. Additionally, it exhibited a recoverable energy density of 0.34 J/cm^3^ and an energy storage efficiency of 81.51% at 50 kV/cm. The P-E loops of (Na_0.2_Bi_0.2_Ba_0.2_Sr_0.2_Ca_0.2_)TiO_3_ ceramic displayed a larger loss when the temperature went above 100 °C. While the (K_0.2_Bi_0.2_Ba_0.2_Sr_0.2_Ca_0.2_)TiO_3_ ceramic maintained its relaxor ferroelectricity and showed a slim characteristic with decreasing P_REM_. These findings suggest that the high-entropy titanate-based oxide containing K/Na and Bi at A-site can be synthesized into dense ceramics with a high dielectric permittivity and relaxor ferroelectric behavior. On the other hand, the compounds with La^3+^ at A-site exhibit less desired properties due to the lack hybridization of 6s^2^ of bismuth and oxygen p-orbital. Thus, this study offers another approach to develop high-performance relaxor ferroelectrics with the desired properties.

## Data Availability

Data are available from the authors upon reasonable request by contacting the corresponding author (Natthaphon Raengthon).
